# Evaluation of lactic acid ferment lysate for the management of oily skin: impact on sebum, hydration, and microbiota

**DOI:** 10.3389/fphys.2026.1791680

**Published:** 2026-06-05

**Authors:** Lei Song, Hongchang Cui, Xingtao Xu, Zhi Duan, Guoqing Meng

**Affiliations:** 1 College of Agriculture and Bioengineering, Heze University, Heze, China; 2 College of Food Science and Engineering, Ocean University of China, Qingdao, China; 3 Qingdao Vland Biotech Group Co., Ltd, Qingdao, China; 4 Qingdao Yisu Biotech INC., Qingdao, China

**Keywords:** cosmetics, microbiota, oily skin, postbiotics, VHProbi^®^ MixP

## Abstract

Oily skin is a prevalent, cosmetically troubling condition. This study investigated the effects of a topical cream with 3% lactic acid ferment lysate VHProbi^®^ MixP for ameliorating oily skin symptoms. A 60-day pilot trial enrolled 42 oily-skin participants, who applied the cream twice daily. Standardized non-invasive tools and imaging systems measured sebum production, porphyrin levels, pore appearance, transepidermal water loss (TEWL), hydration and texture at baseline, day 30 and day 60; 16S rRNA gene sequencing was used for hypothesis-generating analysis of the facial microbiota. By day 60, sebum, porphyrin levels and pore size decreased significantly (31.44%, 44.60% and 25.03% respectively), while hydration and texture improved (63.68% and 38.43% respectively). Hypothesis-generating microbial analysis identified elevated α-diversity and shifted bacterial composition, with reduced *Staphylococcus* spp. and *Cutibacterium* spp. and increased *Streptococcus* spp. Correlation analysis indicated associations between topical application of the cream and reduced sebum secretion, as well as modulated cutaneous microbiota composition. In conclusion, topical application of VHProbi^®^MixP is associated with reduced facial hyperseborrhea, increased epidermal hydration and modulated cutaneous microbial community structure, and may hold potential as an ingredient for oily skin care.

## 1 Introduction

Facial skin can be classified into four main types-oily, dry, neutral, and mixed-based on sebum secretion levels (Youn et al., 2002). Oily skin is characterized by significantly higher sebum production compared to other skin types, as quantified by the mean sebum excretion rate (MSER) (Cunliffe and Shuster, 1969), and is often associated with an imbalance between hydration and sebum output (Qing, 2021). It is a prevalent dermatological concern, clinically presenting as a greasy, shiny, and thickened appearance, along with enlarged pores on the forehead, nose, cheeks, and chin. Individuals with oily skin are more susceptible to psychological distress, including feelings of embarrassment and low self-esteem, due to the increased risk of developing acne vulgaris, seborrheic dermatitis, and uneven skin texture (de Melo and Maia Campos, 2018; Maia Campos et al., 2020; Shuo et al., 2019). As such, this condition has attracted considerable attention in both clinical research and the cosmetic industry.

Sebum plays a pivotal role in maintaining skin homeostasis by reinforcing the skin barrier integrity, defending against microbial invasion, providing natural moisturization, and modulating cutaneous microbiota composition (De Luca and Valacchi, 2010; Feingold, 2007). In healthy adults, normal sebum production averages approximately 1 mg/10 cm² over a three-hour period. Visible oiliness typically occurs when this threshold is exceeded. Sebum secretion is regulated by multiple factors, including endogenous determinants such as hormonal activity, sebaceous gland density, sex, and ethnicity, as well as exogenous influences like age, season, and lifestyle (Géhin et al., 2023; Hameed et al., 2019; Swaney et al., 2023). Excessive sebum production, altered lipid composition, and lipid peroxidation are widely recognized as key contributors to facial oiliness (Vanderwolf et al., 2023). Furthermore, global studies have demonstrated that sebum levels influence the facial microbiota, which in turn shapes the structure and function of the skin’s microbial community (Zheng et al., 2021).

The facial skin microbiota constitutes a complex ecosystem in which bacteria, viruses, and fungi coexist and collectively contribute to skin health (Y. E. Chen et al., 2018; Kim, 2020). Elevated sebum levels promote the proliferation of lipophilic microorganisms, particularly *Cutibacterium* spp., *Staphylococcus* spp., *Pseudomonas* spp., *Actinomyces* spp., and *Corynebacterium* spp (Jiang et al., 2024; Leoty-okombi et al., 2020; Li et al., 2017; Mukherjee et al., 2016; Zheng et al., 2021). To survive in the oligotrophic skin environment, *Cutibacterium* spp. utilizes arginine and other substrates derived from skin proteins and sebum (Bruggemann et al., 2004; Marples et al., 1971; Mukherjee et al., 2016). Moreover, it metabolizes sebaceous lipids into short-chain fatty acids such as acetic and propionic acid, which support bacterial adhesion and colonization (Wilson, 2005). Therefore, investigating the dynamics of the facial skin microbiome is essential for elucidating the underlying mechanisms and progression of oily skin.

Currently, oil-control cosmetics are defined in China’s Cosmetic Product Classification Rules and Catalogue as products that “help reduce sebum secretion and deposition upon application, or minimize the visual appearance of oiliness” (National Medical Products Administration, 2021). For individuals with severe oily skin, clinical interventions such as glycolic acid peels or intradermal botulinum toxin injections can effectively suppress sebum production and prevent complications like acne or seborrheic dermatitis; however, these procedures must be administered in medical settings (Kong and Hwang, 2010; Shuo et al., 2019). Systemic therapies-including adrenal and ovarian androgen inhibitors and 5α-reductase inhibitors-are reserved for severe cases and may carry potential side effects, necessitating supervision by healthcare professionals (Layton et al., 2017; Perez, 2004; Rosette et al., 2019; Searle et al., 2020). With increasing consumer awareness of health and wellness, there is a growing demand for skincare products that are natural, non-irritating, and safe. Microbiome-targeted skincare formulations have gained significant interest, as they offer a non-invasive approach to managing facial oiliness. This study aimed to evaluate whether topical application of the lactic acid ferment lysate VHProbi^®^ MixP could confer beneficial effects for individuals with oily skin. The fermented lysate VHProbi^®^ MixP was produced using three bacterial strains: *Lacticaseibacillus paracasei* E12, *Lactiplantibacillus plantarum* E15, and *Lactiplantibacillus plantarum* O04. In our previous studies, *L. plantarum* E15 displayed antimicrobial activities against *C.acnes* ATCC 11827 and ATCC 6919 and *L*. *paracasei* E12 exhibited antioxidant activity and protective effects *in vitro* experiments (Cui et al., 2022, 2024). *L. plantarum* O04 was isolated from healthy human skin and its genomes was deposited in the National Center for Biotechnology Information with accession number NZ_CP094950 and NZ_CP094951. It effectively inhibits *Corynebacterium kroppenstedtii*, serving to prevent and ameliorate facial redness and type I rosacea with prominent improvement effects. As an exploratory pilot investigation, the present study employed a single-arm, unblinded design without a vehicle or active comparator group. This experimental design limits the ability to draw definitive causal inferences between VHProbi^®^ MixP and any observed changes in oily skin parameters, and potential placebo effects cannot be excluded. Thus, the findings from this study are intended to generate preliminary evidence only, and well-designed double-blind, placebo-controlled randomized trials will be required for subsequent confirmatory assessment of the ferment lysate’s potential effects.

## 2 Materials and methods

### 2.1 Preparation of ferment lysate

Three strains of *L. plantarum* E15, *L*. *paracasei* E12 and *L. plantarum* O04 were cultured in a fermentation medium consisting of 5% collagen, 2% molasses, 0.3% (NH_4_)_2_HPO_4_, 0.1% peptone, and distilled water to a final volume of 1000 mL. Fermentation was conducted in a 30 L bioreactor (NCBIO^®^, FUS-30L) at 37 °C for 48 hours. After completion of fermentation, the cultures were homogenized via high-pressure homogenization (SCIENTZ^®^ Scientz-150) at 1000 bar for three consecutive cycles. The individual lysates were then combined in equal proportions to formulate VHProbi^®^ MixP, which was used in subsequent experiments (Cui et al., 2023).

### 2.2 Cream formulation

The cream formulation consisted of the following ingredients: Aqua (q.s. to 100%), 5% dextran, 4% carbomer, 3% hydroxyethylcellulose, 3% VHProbi^®^ MixP, 2% xanthan gum, 0.8% phenoxyethanol, 0.5% ethylhexylglycerin, 0.2% potassium sorbate, 0.2% disodium ethylenediaminetetraacetate (EDTA), 0.3% sodium citrate, and 0.1% sodium hydroxide. Except for VHProbi^®^ MixP, all other components functioned as thickeners, preservatives, and chelating agents within the formulation.

### 2.3 Clinical studies

#### 2.3.1 Subject recruitment

Forty-two volunteers (male and female) were enrolled in this study following evaluation against the predefined inclusion and exclusion criteria (**Table 1**). All completed the 60-day study without dropouts, missing visits, or exclusions due to adverse events or non-compliance. Thus, the full cohort of 42 participants was included in all skin parameter and microbiome analyses across all time points. In accordance with the Specification for Cosmetic Efficacy Claim Evaluation and the Safety and Technical Standards of Cosmetics (2015 Edition) issued by the National Medical Products Administration of China (National Medicine Products Administration, 2021a, 2021b), clinical trial registration was not required. All procedures adhered to the principles outlined in the Declaration of Helsinki (Goodyear et al., 2007).

**Table 1:** Inclusion and exclusion criteria for the study.

Inclusion criteria	Exclusion criteria
Male or female participants aged 18 to 45 years.	Known hypersensitivity or allergy to any ingredient of the test product.
Signed informed consent form.	Pregnant or breastfeeding.
No participation in other clinical studies currently or in the past 6 months	Facial disease or any facial cosmetic procedures within 3 months.
Ability to follow the instructions from investigators and return to study center within the required time.	Use of esthetic and/or dermatological treatments in the areas of study product application within 21 days.
Fitzpatrick phototype classification I–VI.	Significant psychiatric or psychological disorders that may affect compliance or ability to provide reliable self-assessment.
Subjects with oily skin.	Use of antihistamines, immunosuppressants, nonsteroidal anti-inflammatory drugs (NSAIDs), local anesthetics, or corticosteroids within 14 days or during this study.
Not receiving any cosmetic treatments/drugs that might interfere with current study	Acute exacerbation of a dermatosis, systemic or dermatological infection, psychiatric, or malignant diseases.

Participants with a skin phototype classified as I–VI on the Fitzpatrick scale (Sachdeva, 2009), who self-reported oily skin, and who had sebum levels ≥ 50, were included in the study. Detailed information on all subjects is provided in **Supplementary Table 1**. To minimize potential confounding factors, two trained staff members oversaw the recruitment process, ensuring strict adherence to the predefined inclusion and exclusion criteria. Data collection and analysis were performed independently by two personnel to ensure data integrity and accuracy. An independent monitor supervised the entire study procedure to ensure compliance with the established protocols.

#### 2.3.2 Study procedures and sampling

Participants applied a thin layer of the investigational cream (equivalent to 1 g) twice daily-once in the morning and once in the evening-for a duration of 60 days. During the assessment periods, participants were instructed to refrain from facial cleansing, makeup application, or sunscreen use for at least six hours prior to each evaluation. They were also required to rest for 20 minutes in the assessment room, which was maintained at a controlled temperature of 24-25 °C and relative humidity of 50-60%, to ensure skin acclimatization before measurements. Dermatological parameters, including sebum levels, skin hydration, and transepidermal water loss (TEWL), were measured using the Z0010M Sebumeter Probe, S0033 Moisture Probe, and S0083 TEWL Probe of the CK-MPA^®^ 224 system (CK-MPA10, Courage + Khazaka Electronic GmbH, Germany), respectively. Assessments of porphyrin fluorescence, pore appearance, and skin texture were conducted using the VISIA^®^-CR imaging system (Canfield Scientific Inc., USA). All objective measurements were performed at three time points: baseline (day 0), mid-treatment (day 30), and final evaluation (day 60). The Efficacy Self-Assessment Questionnaire for oily skin was administered at baseline and endpoint.

For microbial sample collection, a defined 4 cm × 4 cm area on the cheek was sampled by firmly rubbing with a sterile swab pre-moistened in a sterile wetting solution (0.9% NaCl and 0.1% Tween 20; Sigma^®^). The swab tips were then aseptically excised and immediately stored at -80 °C until DNA extraction. To minimize cross-contamination, all sampling procedures were performed adjacent to an ignited alcohol lamp under aseptic conditions, and new sterile gloves were worn for each participant. Microbial samples were collected from all 42 participants at both baseline (Day 0) and study endpoint (Day 60), with no missing samples. Each participant contributed two paired samples (one at each time point), resulting in a total of 84 paired samples for microbiome analyses.

#### 2.3.4 DNA extraction and 16S V3-V4 amplicon sequencing

Total genomic DNA was extracted from cheek swab samples using the TIANGEN^®^ TIANamp Bacteria DNA Kit according to the manufacturer’s instructions. The quality and concentration of the extracted DNA were assessed by 1.0% agarose gel electrophoresis and a NanoDrop 2000 spectrophotometer (Thermo Fisher Scientific, USA). Purified DNA was stored at -20 °C until further use for PCR amplification.

The V3-V4 hypervariable region of the 16S rRNA gene was amplified using the primer pairs 338F (5’-ACTCCTACGGGAGGCAGCAG-3’) and 806R (5’-GGACTACHVGGGTWTCTAAT-3’) on a T100 Thermal Cycler (Bio-Rad, USA) (López-Aladid et al., 2023). Following amplification, all amplicon products were purified using the PCR Clean-Up Kit (YuHua, Shanghai, China) and quantified using a Qubit 4.0 fluorometer (Thermo Fisher Scientific, USA) in accordance with the manufacturers’ protocols.

Sequencing libraries were constructed using the NEXTFLEX^®^ Rapid DNA-Seq Kit (Illumina, USA) and evaluated for quality and size distribution using an Agilent Bioanalyzer 2100 system (Agilent Technologies, USA) (Zhai et al., 2018). Qualified libraries were subsequently sequenced on the Illumina NovaSeq 2000 platform (Illumina, USA) following standard sequencing protocols provided by Majorbio Bio-Pharm Technology Co. Ltd. (Shanghai, China).

#### 2.3.5 Data processing

The paired-end raw sequencing reads were subjected to quality control using fastp software (version 0.23.2) (S. Chen, 2023), and sequence assembly was performed using FLASH software (version 1.2.11) (Magoč & FLASH, 2011). Operational taxonomic units (OTUs) were clustered at a 97% similarity threshold using UPARSE software (version 10.0) (Edgar, 2013), followed by the removal of chimeric sequences. To minimize the impact of variable sequencing depth on downstream analyses, all samples were normalized to the lowest read count via subsampling using Mothur software (version 1.30.2) (Schloss et al., 2009). Taxonomic annotation of OTUs was conducted using the RDP classifier (version 11.5) (Wang et al., 2007) against the SILVA 16S rRNA gene database (release 138.2) (Quast et al., 2012) with a minimum confidence threshold of 70%. The microbial community composition of each sample was statistically analyzed across multiple taxonomic levels.

All data analyses were performed using the online MajorBio Cloud Platform (https://cloud.majorbio.com) as follows (Ren et al., 2022): Alpha (α) diversity indices, including Chao1, Shannon, and Simpson, were calculated using Mothur software. The Wilcoxon rank-sum test was applied to assess intergroup differences. Beta (β) diversity among samples was evaluated through principal coordinates analysis (PCoA) based on the Bray–Curtis distance metric, implemented with the Vegan package (v2.6-8) (Oksanen et al., 2007). ANOSIM (Analysis of Similarities) was also conducted to determine the statistical significance of microbial community structure differences between sample groups. LEfSe (Linear Discriminant Analysis Effect Size) analysis (http://huttenhower.sph.harvard.edu/lefse; LEfSe analysis was conducted with nominal P < 0.05 and LDA score > 2 as preliminary screening criteria only, with no rigorous statistical control for multiple comparisons to identify taxa exhibiting significant abundance differences from phylum to genus level across groups (Segata et al., 2011). Spearman’s rank-correlation coefficient was used for exploratory correlation analysis (|r| > 0.3, P < 0.05), with results visualized via heatmap representation for hypothesis generation only (De Winter et al., 2016). All microbiome analyses, including α-diversity index comparisons, LEfSe analysis and Spearman’s rank-correlation analysis, were pre-defined as hypothesis-generating. No false discovery rate (FDR) correction was applied across taxa or correlation pairs for any microbiome analysis.

#### 2.3.6 Statistical analysis

Data were initially processed using EpiData version 3.1 and subsequently exported to SPSS version 26.0 (IBM SPSS Statistics, USA) for further statistical analysis. Primary and secondary endpoints for skin biophysical parameters were pre-specified prior to study initiation: (1) Primary endpoint: The change in sebum level from baseline (Day 0) to the final evaluation (Day 60); (2) Secondary primary endpoint: The change in transepidermal water loss (TEWL) from baseline (Day 0) to Day 60; (3) Secondary endpoints: Changes in porphyrin fluorescence, pore appearance, skin hydration and skin texture from baseline to Day 30/Day 60. All skin parameter analyses were framed as exploratory, and NO multiple comparison correction was applied across the six skin parameters or two post-baseline time points (Day 30 and Day 60). The normality of data distribution was assessed using the one-sample Kolmogorov-Smirnov test. Continuous variables are reported as mean ± standard deviation (SD). For skin parameters, changes in measurements collected at baseline (Day 0), mid-treatment (Day 30), and endpoint (Day 60) were analyzed using repeated-measures ANOVA. For microbiome α-diversity indices, differences between time points were analyzed using the Wilcoxon signed-rank test. Statistical significance reported herein refers to nominal *P* values only: *P* < 0.05 was considered nominally statistically significant, with *P* < 0.01 considered nominally highly significant. Graphical representations were generated using GraphPad Prism version 8.0.

## 3 Results

### 3.1 Skin parameters

All 42 enrolled participants completed the study with no dropouts, missing visits, or excluded data points. Thus, n=42 for all skin parameter analyses at baseline (Day 0), Day 30, and Day 60. Results are exploratory, with nominal *P* values reported and no multiple comparison correction applied across parameters or time points.

#### 3.1.1 Sebum levels

**Figure 1:**
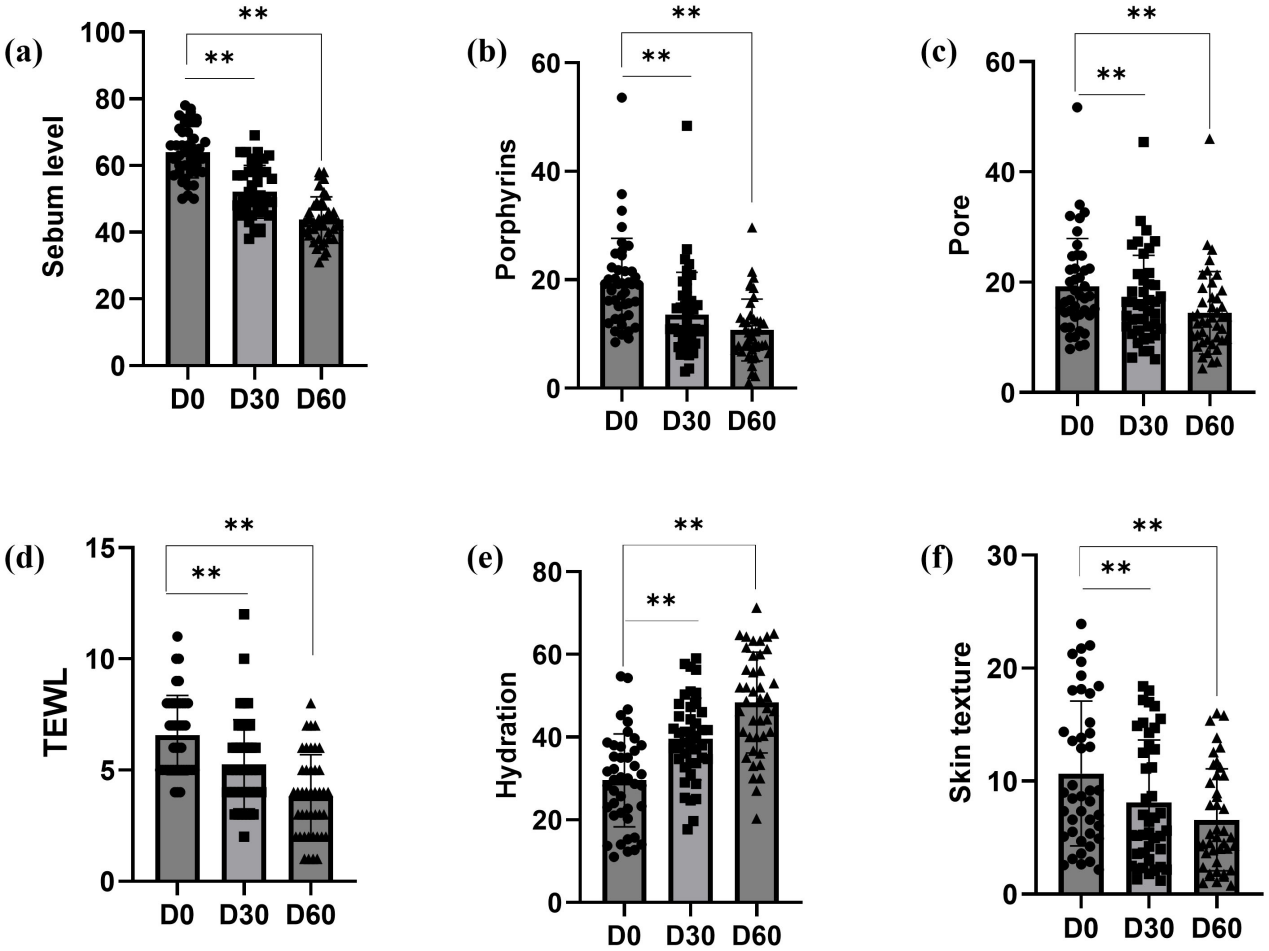
Detailed scores for six indicators related to the alleviation of facial oiliness: **(a)** sebum level, **(b)** porphyrin fluorescence, **(c)** pore appearance, **(d)** transepidermal water loss (TEWL), **(e)** skin hydration, and **(f)** skin texture. D0: Day 0; D30: Day 30; D60: Day 60; **P < 0.01. Date was presented with mean and standard deviation. n=42 for all time points; no dropouts, missing visits, or excluded participants; least−squares means from repeated−measures ANOVA with Sidak correction.

Detailed results on sebum levels for individuals from baseline to endpoint are presented in **Supplementary Table 2**. As shown in **Figure 1a**, the mean sebum level decreased from 64.00 at baseline (Day 0) to 52.14 at Day 30, and further to 43.88 at Day 60, representing reductions of 18.53% and 31.44%, respectively. Nominally highly significant differences in sebum levels were observed at Day 30 and Day 60 compared with baseline (nominal *P* < 0.01). These exploratory findings indicate that the cream containing VHProbi^®^ MixP is associated with reduced skin sebum levels in the study cohort.

#### 3.1.2 Skin porphyrins

**Figure 2:**
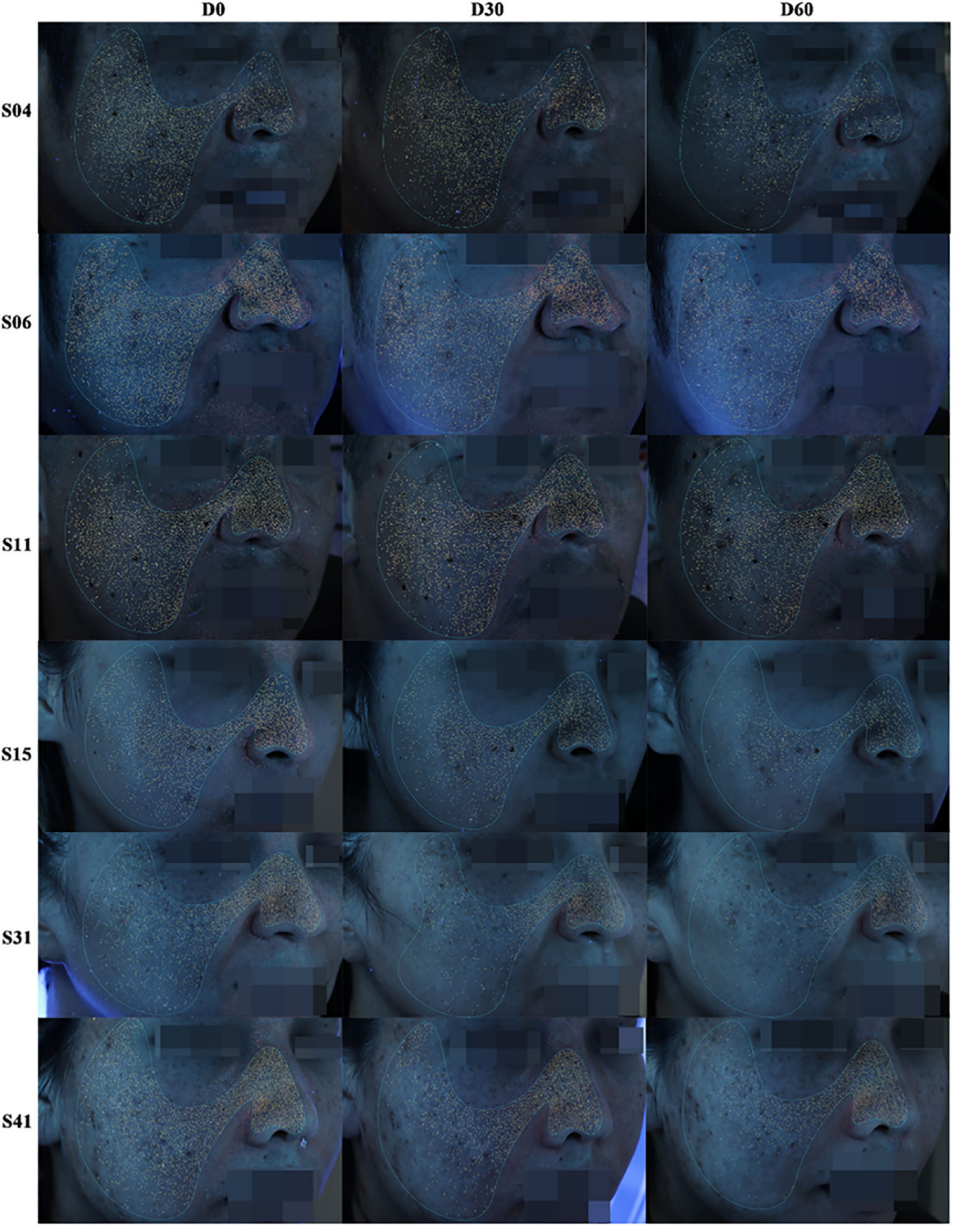
Temporal changes in facial porphyrin fluorescence levels in representative subjects over a 60-day period. Images were captured using the VISIA^®^-CR imaging system. The discrete orange-yellow fluorescent spots within the highlighted regions of interest represent porphyrins levels. A progressive reduction in the density and intensity of these fluorescent spots is observed from Day 0 (D0) to Day 30 (D30) and Day 60 (D60).

Detailed results on porphyrin levels from baseline to endpoint are presented in **Supplementary Table 2**. As shown in **Figure 1b**, the mean porphyrin level decreased from 19.35 at baseline (Day 0) to 13.53 at Day 30, and further to 10.72 at Day 60, representing reductions of 30.08% and 44.60%, respectively. Nominally highly significant differences were observed at Day 30 and Day 60 compared with baseline (nominal *P* < 0.01).Representative photographic evaluations (**Figure 2**) confirmed a progressive decline in porphyrin fluorescence over the 60-day period, an exploratory finding suggesting reduced sebaceous gland activity on the cheeks in association with cream application.

#### 3.1.3 Skin pores

Detailed scores on skin pore metrics from baseline to endpoint are presented in **Supplementary Table 2**. As shown in **Figure 1c**, the mean pore score decreased from 19.26 at baseline (Day 0) to 16.95 at Day 30, and further to 14.22 at Day 60, representing reductions of 11.99% and 25.03%, respectively. Nominally highly significant differences were observed at Day 30 and Day 60 compared with baseline (nominal *P* < 0.01).

**Figure 3:**
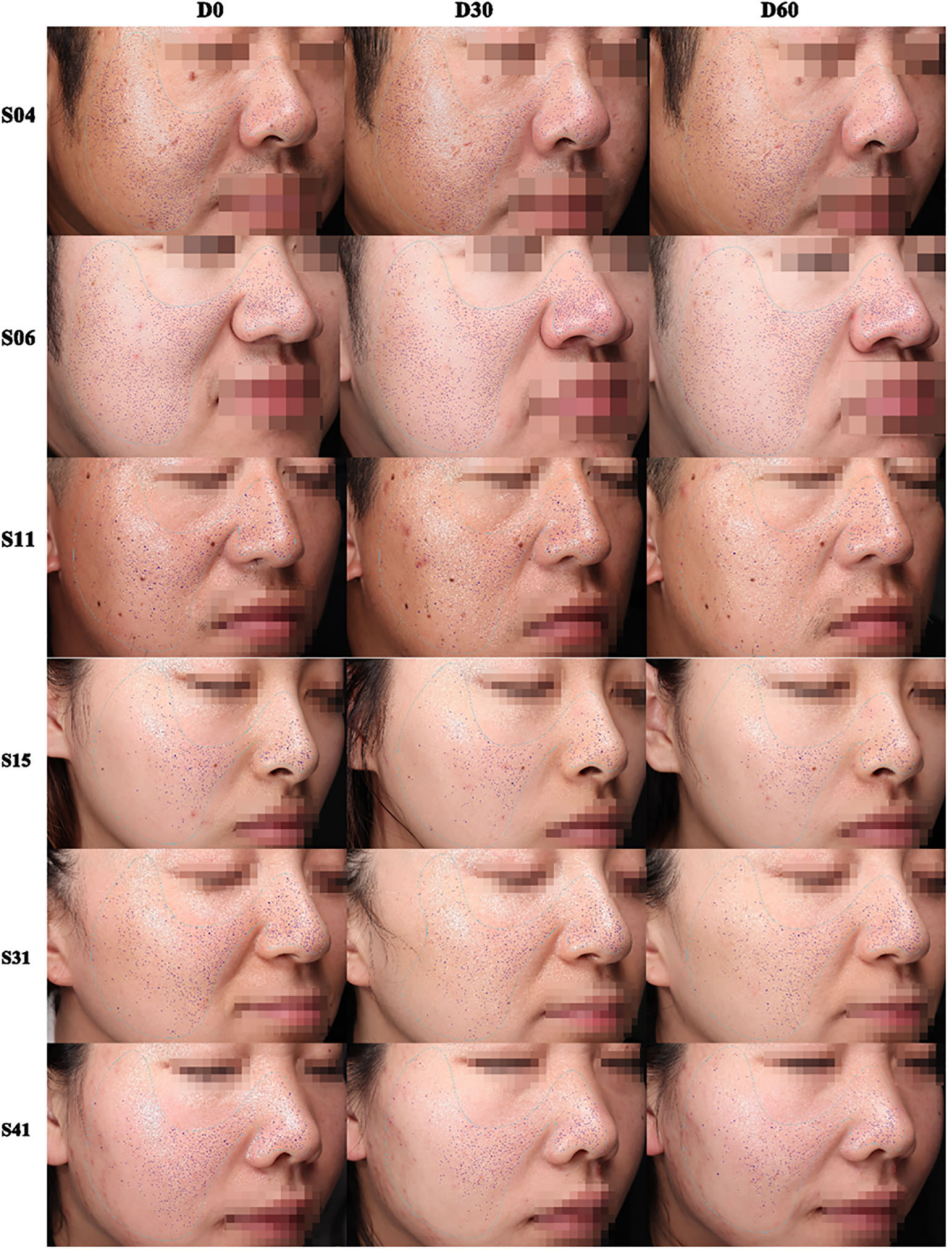
Visual representation of facial pore changes over a 60-day period. Images were captured using the VISIA^®^-CR imaging system. The purple-colored overlay markers indicate individual pores automatically detected and mapped by the system’s feature-recognition software based on skin surface topography and contrast. A progressive reduction in the visibility, size, and density of pores is observed within the designated regions of interest from Day0 (D0) to Day 30 (D30) and Day 60 (D60).

Visual representation of pore changes (**Figure 3**) showed a marked reduction in visible pore number and density in key facial regions at Day 60, an exploratory finding associated with cream application.

#### 3.1.4 Detection of TEWL

Detailed results on TEWL from Day 0 to Day 60 are presented in **Supplementary Table 2**. As shown in **Figure 1d**, the mean TEWL value decreased from 6.57 at baseline (Day 0) to 5.26 at Day 30, and further to 3.83 at Day 60, representing reductions of 19.94% and 41.70%, respectively. Nominally highly significant differences in TEWL were observed at both Day 30 and Day 60 compared with baseline (nominal *P* < 0.01). These exploratory findings suggest an association between VHProbi^®^ MixP application and enhanced skin water retention capacity.

#### 3.1.5 Skin hydration

Detailed changes in skin hydration levels from Day 0 to Day 60 are presented in **Supplementary Table 2**. As shown in **Figure 1e**, the mean skin hydration level increased from 29.54 at baseline (Day 0) to 39.54 at Day 30, and further to 48.35 at Day 60, representing increases of 33.85% and 63.68%, respectively. Compared with baseline, facial hydration showed a significant increase at both Day 30 and Day 60 (P < 0.01). Nominally highly significant increases in facial hydration were observed at both Day 30 and Day 60 compared with baseline (nominal *P* < 0.01). This exploratory finding demonstrates a progressive improvement in hydration levels over the 60-day period, suggesting enhanced skin moisture retention in association with cream application.

#### 3.1.6 Skin texture

Detailed changes in skin texture scores from Day 0 to Day 60 are presented in **Supplementary Table 2**. As shown in **Figure 1f**, the mean skin texture score decreased from 10.67 at baseline (Day 0) to 8.10 at Day 30, and further to 6.57 at Day 60, representing reductions of 24.09% and 38.43%, respectively. Nominally highly significant improvements in skin texture were observed at Day 30 and Day 60 compared with baseline (nominal *P* < 0.01). This exploratory finding indicates enhanced skin smoothness in association with prolonged cream application.

#### 3.1.7 Self-assessment questionnaire

Participants were asked to rate their perceptions of reduced facial greasiness, improved skin smoothness, refined texture, and decreased itchiness, among other oily skin-related concerns. Detailed self-assessment questionnaire scores are presented in **Supplementary Table 3**. More than 81% of subjects reported good to excellent improvements in facial oiliness, smoothness, fineness, and itchiness, with self-reported outcomes consistent with the exploratory instrumental assessments of skin parameters (no multiple comparison correction applied).

### 3.2 Bacterial composition analysis

Microbiome analyses included 84 paired samples, with all 42 participants contributing one sample at baseline (Day 0) and one sample at Day 60 (no missing samples). Thus, the dataset consisted of 42 matched participant pairs (Day 0 vs. Day 60), ensuring the paired nature of the design. The raw data derived from high-throughput sequencing have been deposited in the NCBI (National Center for Biotechnology Information) SRA (Sequence Read Archive) database under accession number PRJNA1210067. After processing and filtering of 5,551,421 raw reads, a total of 5,320,206 validated sequencing reads were generated from 84 cheek skin samples, with 3,118 operational taxonomic units (OTUs) identified across 38 phyla, 281 orders, 502 families, 1,179 genera, and 2,143 species.

#### 3.2.1 Microbial diversity analysis

**Figure 4:**
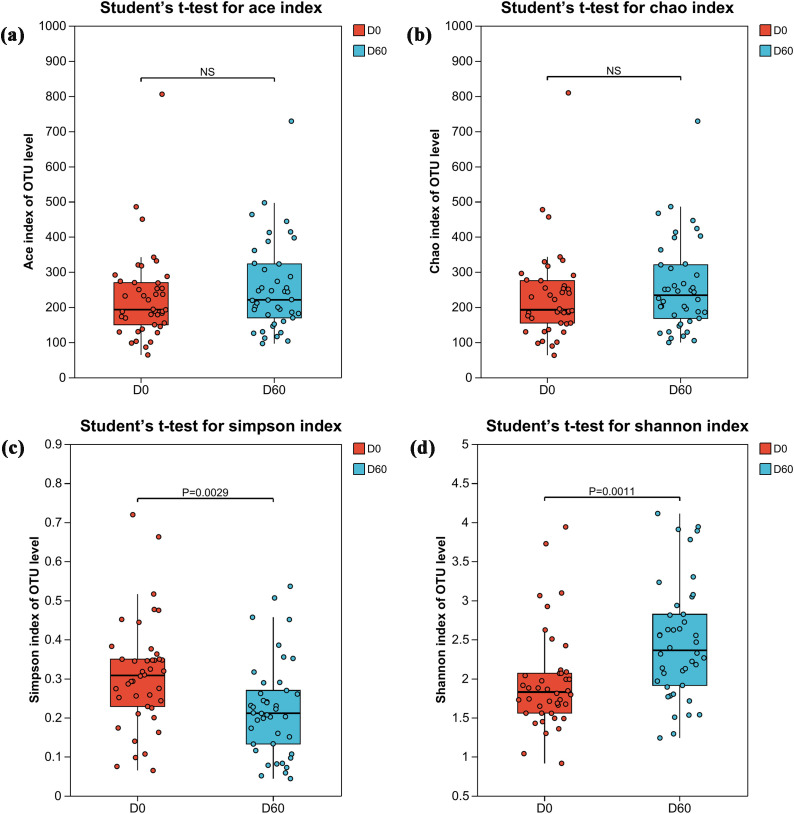
Box plots of ace **(a)**, Chao **(b)**, Simpson **(c)**, and Shannon **(d)** indices for bacterial 16S rRNA gene diversity in cheek skin between Day 0 (D0) and Day 60 (D60). The significance of differences between sampling groups is indicated by P value. NS, not significant.

Ace, Chao, Simpson, and Shannon indices were used to explore the evenness and richness of facial cheek skin microbiota between baseline (Day 0) and endpoint (Day 60) (**Figure 4**). Ace and Chao indices were observed to be higher at Day 60 compared to Day 0 (nominal *P* > 0.05, Wilcoxon signed-rank test, paired), while nominally highly significant differences were identified in the Simpson and Shannon indices between Day 0 and Day 60 (nominal *P* < 0.01, Wilcoxon signed-rank test, paired). These hypothesis-generating findings identify preliminary changes in α-diversity in the facial cheeks of individuals with oily skin in association with VHProbi^®^ MixP application.

**Figure 5:**
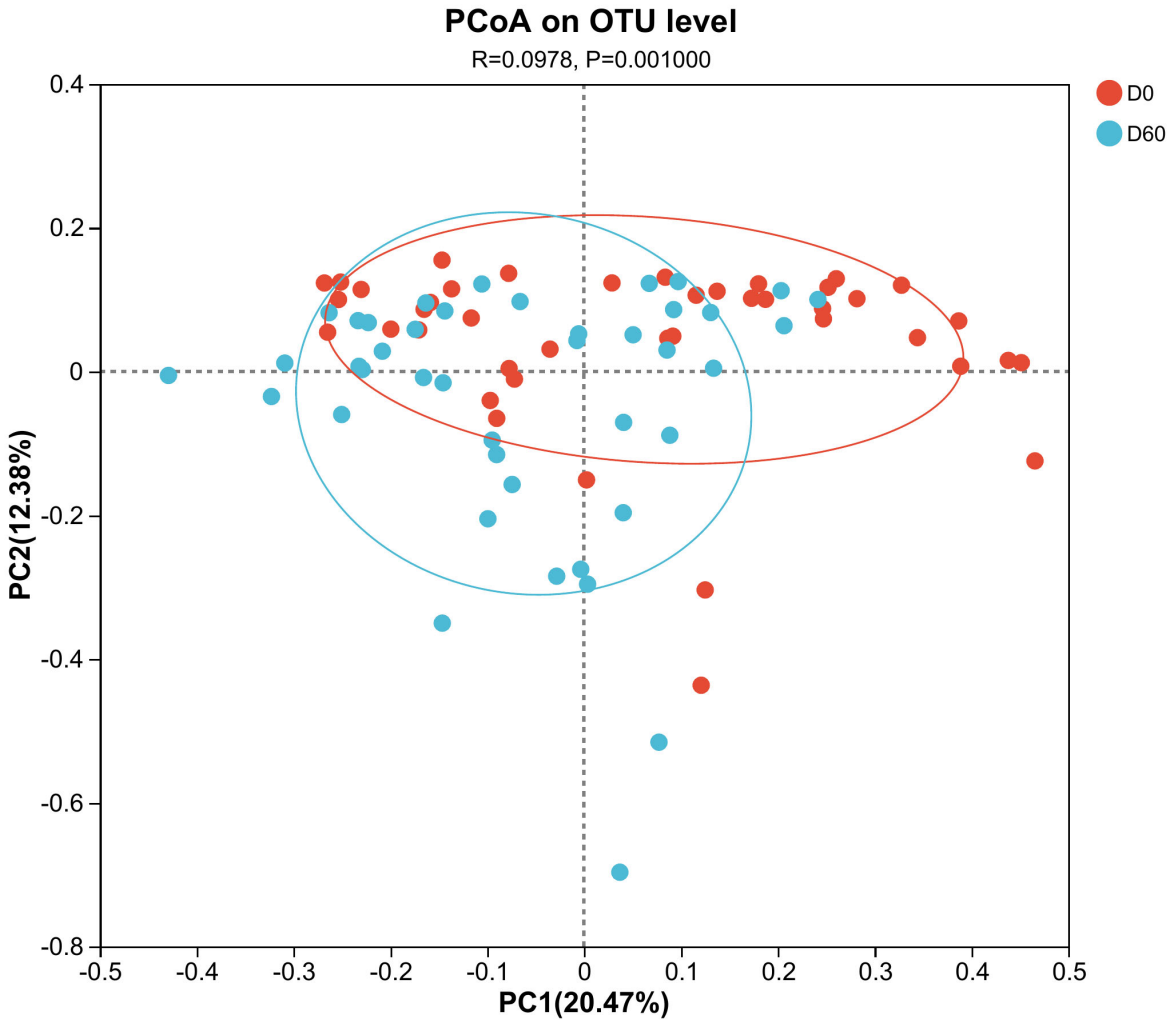
PCoA (principal coordinates analysis) score plot at the OTU level before and after cream application. Distance metric was calculated with Bray_cutis. Red dots represent bacterial communities from samples collected at Day 0 (D0); light blue dots represent bacterial communities from samples collected at Day 60 (D60).

PCoA combined with ANOSIM analysis were conducted at the OTU level (**Figure 5**). The PCoA plot revealed a substantial overlap between the D0 and D60 groups, indicating that the overall microbial community structure remained relatively stable throughout the study. Although ANOSIM identified a statistically significant difference (P = 0.001), the low *R*-statistic (R = 0.0978) suggested that the magnitude of this difference was minor, with the community compositions being highly similar between the two time points. Notably, the D60 group exhibited a wider distribution (greater within-group dispersion) across the PC1 and PC2 axes compared to D0, suggesting a subtle increase in microbial community variability following the 60-day treatment, rather than a distinct compositional shift.

#### 3.2.2 Microbial composition analysis

**Figure 6:**
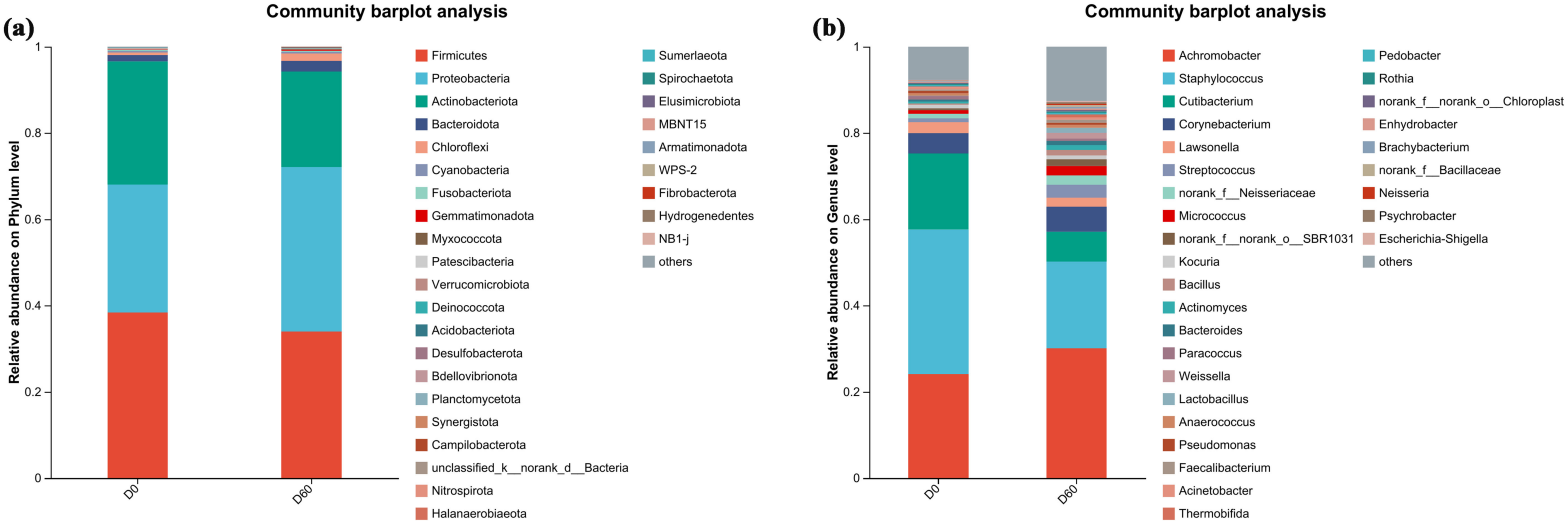
Top 30 bacterial taxa at the **(a)** phylum and **(b)** genus levels in terms of relative abundance between the starting point and endpoint. D0, Day 0; D60, Day 60.

Stacked bar charts of the top 30 most abundant taxa at the phylum and genus levels were generated to explore relative abundance changes. At the phylum level (**Figure 6a**), the relative abundance of *Firmicutes* (38.35% vs. 33.97%) and *Actinobacteriota* (28.55% vs. 22.18%) was observed to decrease at Day 60 compared to Day 0, while *Proteobacteria* (29.69% vs. 38.11%), *Bacteroidota* (1.48% vs. 2.41%), and *Chloroflexi* (0.56% vs. 1.76%) showed increased relative abundance. At the genus level (**Figure 6b**), *Achromobacter* (24.07% vs. 30.12%), *Corynebacterium* (4.71% vs. 5.83%), and *Streptococcus* (0.85% vs. 3.00%) were observed to increase in relative abundance, while *Staphylococcus* (33.55% vs. 20.07%), *Cutibacterium* (17.64% vs. 6.92%), and *Lawsonella* (2.54% vs. 2.05%) showed reduced relative abundance from baseline to endpoint. These are hypothesis-generating observations of taxonomic abundance shifts associated with VHProbi^®^ MixP application.

#### 3.2.3 Species difference analysis

**Figure 7:**
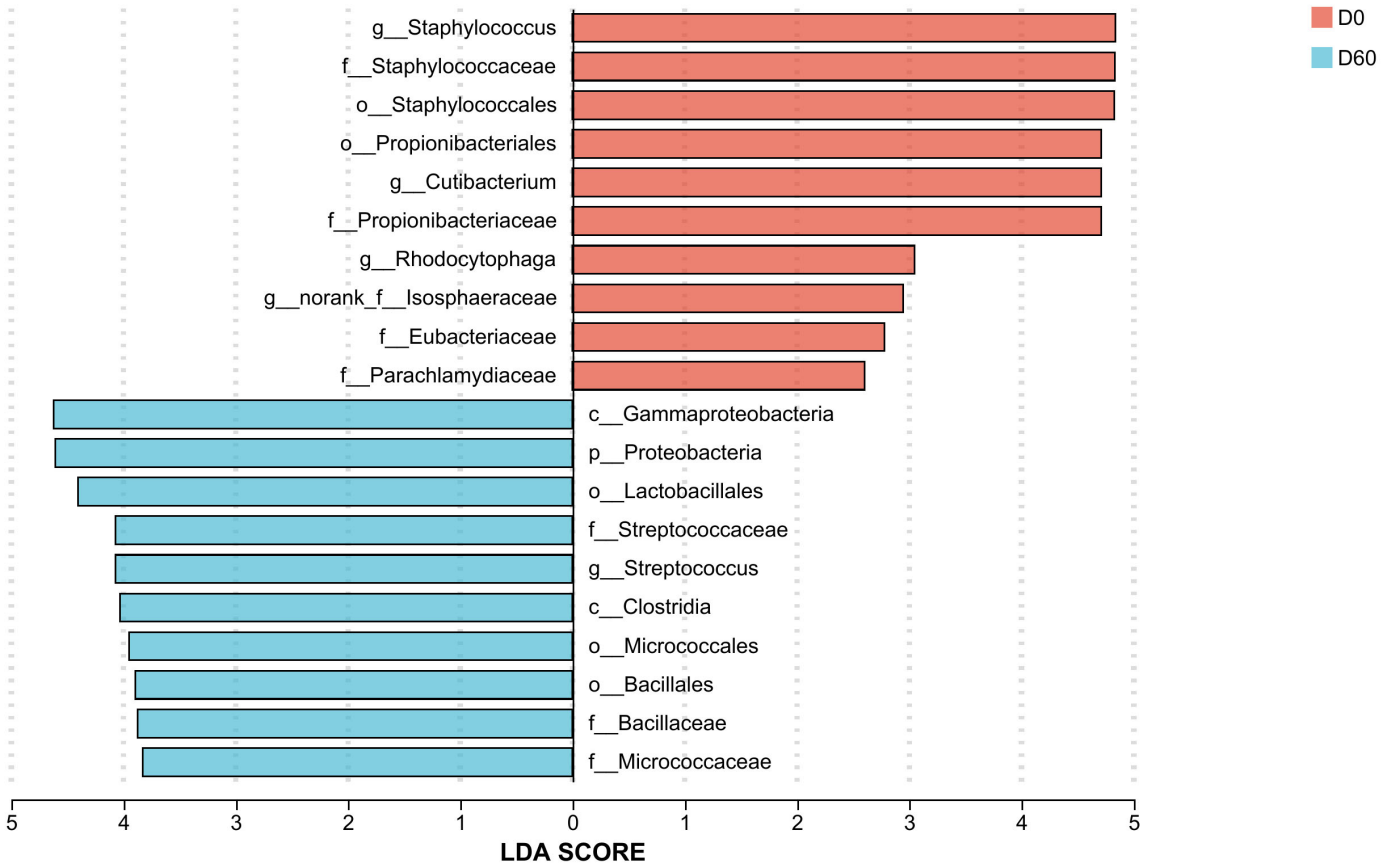
Linear discriminant analysis effect size (LEfSe) of bacterial groups with differential abundance between the D0 and D60 groups. Significance levels for LEfSe were set as P < 0.05, and only bacterial groups with a linear discriminant analysis (LDA) score > 2 are displayed. D0: Day 0; D60: Day 60.

LEfSe analysis (nominal *P* < 0.05, LDA score > 2, no FDR correction) was performed to identify differentially abundant bacterial groups from phylum to genus level (**Figure 7**; **Supplementary Figure 1**). Nominally significant differences in bacterial composition abundance were observed at both phylum and genus levels: *Staphylococcus*, *Cutibacterium*, and *Rhodocytophaga* were enriched in the D0 group, while *Streptococcus* (genus) and *Lactobacillales* (order) showed significantly higher relative abundance in the D60 group (nominal P < 0.05). These hypothesis-generating findings identify key taxa that may be associated with VHProbi^®^ MixP application in oily skin, warranting further confirmatory investigation.

#### 3.2.4 Microbial correlation analysis

**Figure 8:**
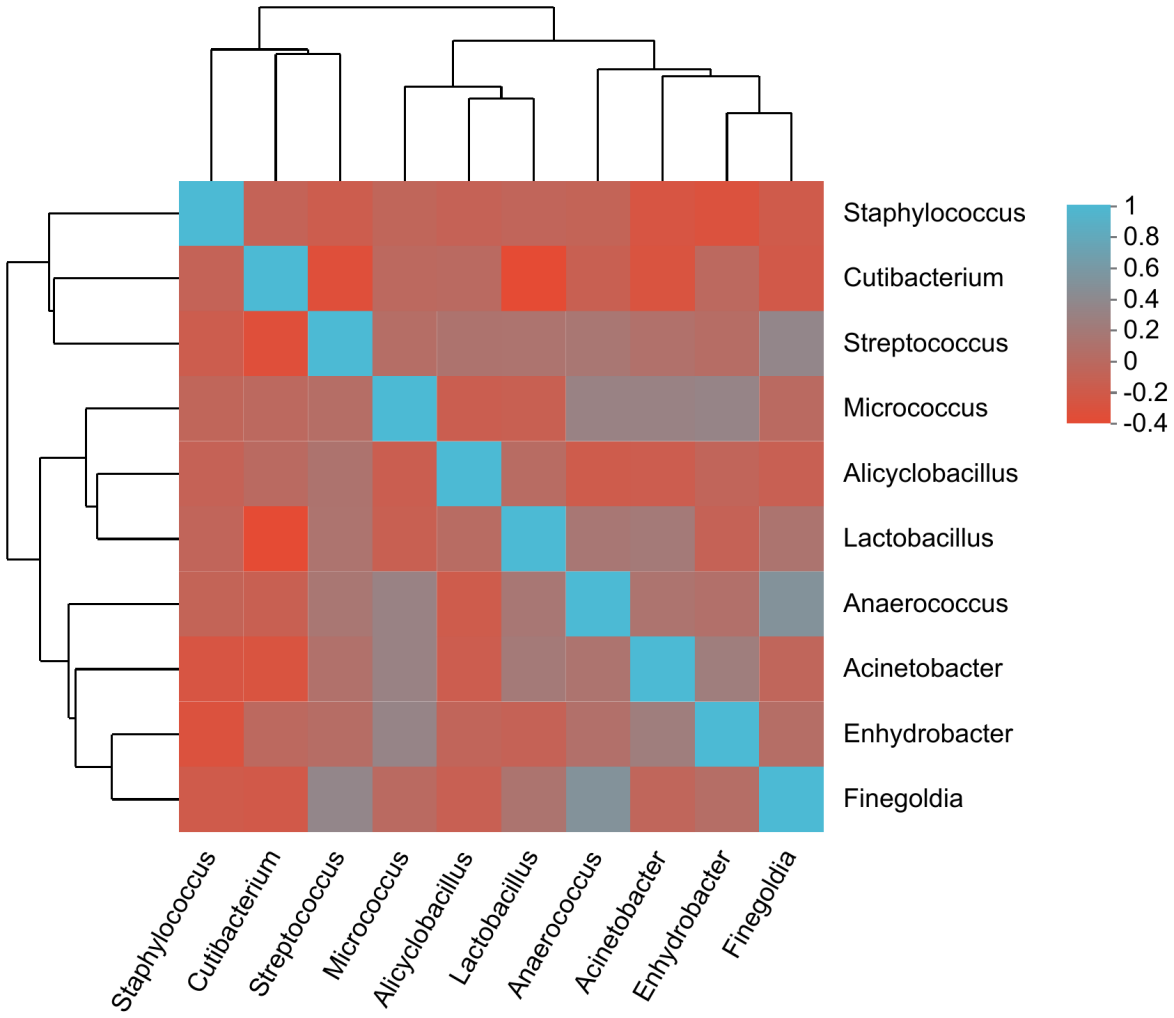
Spearman correlation analysis of bacterial genera with significant differences between Day 0 and Day 60.

Spearman correlation analysis (nominal *P* < 0.05, no FDR correction) of bacterial genera with nominally significant abundance differences identified preliminary pairwise correlations (**Figure 8**): *Streptococcus* showed a weak positive correlation with *Finegoldia* (r = 0.38, nominal P < 0.01), *Cutibacterium* showed a weak negative correlation with *Lactobacillus* (r = -0.33, nominal P < 0.01), and *Micrococcus* showed weak positive correlations with *Anaerococcus*, *Acinetobacter*, and *Enhydrobacter* (all r ≥ 0.33, nominal P < 0.01). *Anaerococcus* also showed a weak positive correlation with *Finegoldia* (r = 0.52, nominal P < 0.01). No other nominally significant correlations were observed among these genera.

Spearman correlation analysis (no FDR correction) between α-diversity and skin parameters identified no nominally significant associations (all nominal P > 0.05, **Table 2**), a hypothesis-generating finding suggesting no obvious link between α-diversity changes and the measured skin biophysical parameters in this study cohort.

**Table 2:** Spearman correlation results between α-diversity and skin parameters.

Parameters	Ace		Chao		Shannon		Simpson	
	Rho	*p-*value	Rho	*p-*value	Rho	*p-*value	Rho	*p-*value
Sebum	0.20	0.07	0.21	0.05	0.02	0.84	0.05	0.68
Porphyrins	0.09	0.40	0.11	0.33	-0.07	0.51	0.09	0.41
Pore	-0.16	0.14	-0.14	0.20	-0.20	0.07	0.17	0.12
TEWL	0.17	0.13	0.17	0.12	-0.02	0.85	0.16	0.15
Hydration	-0.18	0.10	-0.19	0.09	0.01	0.91	-0.09	0.44
Texture	0.11	0.31	0.13	0.22	0.01	0.92	-0.02	0.88

**Figure 9:**
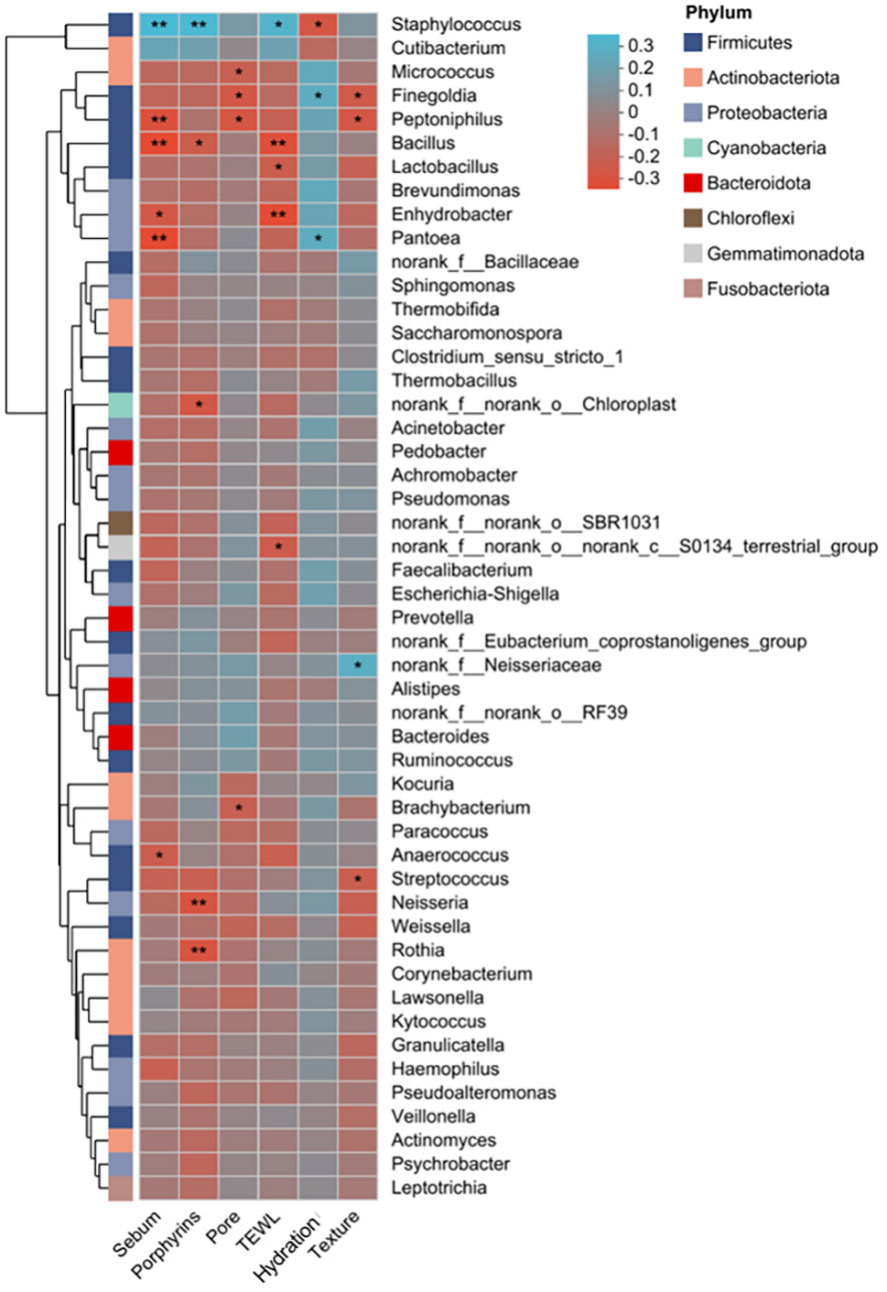
Spearman correlation heatmap between skin parameters and bacterial genera. The relative abundance of the top 50 genera is shown. Asterisks denote significant difference in the heatmap. *P < 0.05; **P < 0.01.

As illustrated in **Figure 9**, spearman correlation analysis (no FDR correction) between skin parameters and the top 50 most abundant microbial genera identified a weak positive correlation between *Staphylococcus* and sebum levels (r = 0.30, nominal P < 0.01) as well as porphyrin fluorescence (r = 0.30, nominal P < 0.01) and TEWL (r = 0.27, nominal P < 0.05). Conversely, a significant negative correlation was observed between *Staphylococcus* and hydration (r = -0.27, nominal P < 0.05). In terms of pore and texture, no statistically significant correlation was identified (P > 0.05). Likewise, no statistically significant correlation was identified between *Cutibacterium* and skin parameters (P > 0.05). This hypothesis-generating finding identifies a preliminary association between *Staphylococcus* abundance and key oily skin parameters in this study.

## 4 Discussion

Facial skin appearance plays a pivotal role in social interaction and perception. Clear, radiant skin is often associated with youth and vitality—attributes that are highly valued across many cultures (Voegeli et al., 2021). The most distinctive feature of oily skin compared to other skin types is excessive sebum production on the skin surface, coupled with low water content, leading to an imbalanced oil-to-water ratio (Kuang et al., 2025). Furthermore, sebum levels influence the composition and abundance of the cutaneous microbial community, with lipophilic bacterial genera being particularly predominant in sebaceous areas such as the facial T-zone including cheeks, forehead, and upper back (Condrò et al., 2022). Exploring the mechanisms underlying skin greasiness has driven the development of promising cosmetic ingredients for individuals seeking anti-oil skincare solutions. The use of metabolic byproducts or lysates derived from probiotic fermentation as functional skincare ingredients has a long-standing history in the cosmetics industry.

It is critical to note the key statistical design considerations of this pilot investigation: (1) primary and secondary endpoints for skin parameters were pre-specified, but no multiple comparison correction was applied across the six skin parameters or two post-baseline time points, with all skin parameter results framed as exploratory; (2) all microbiome analyses (α-diversity, LEfSe, Spearman correlations) were pre-defined as hypothesis-generating, with no FDR correction applied across taxa or correlation pairs, and LEfSe analysis relied on nominal *P* < 0.05 + LDA > 2 as preliminary screening only (no rigorous statistical control). Thus, all observations in this study are associative and exploratory/hypothesis-generating, with no definitive causal inferences or statistical confirmations possible.

In the present study, a pilot investigation was conducted to evaluate whether a cream containing VHProbi^®^ MixP could benefit individuals with oily facial skin. Our results confirmed a significant reduction in sebum secretion on cheek skin after a 60-day intervention compared to baseline measurements at Day 0. Images captured by the VISIA^®^-CR system under orange light mode were analyzed using specialized software to quantify porphyrin fluorescence, a recognized indicator of sebum accumulation. Both porphyrin levels and pore scores were significantly reduced at Day 60 relative to baseline. Additionally, participants’ skin barrier function improved significantly, as evidenced by decreased TEWL and increased hydration indices, indicating restoration of the skin’s oil-to-water balance. Moreover, self-reported questionnaire outcomes aligned closely with instrumental assessments, confirming that the majority of participants perceived the cream containing VHProbi^®^ MixP correlating with addressing their oily skin concerns.

Sebum is essential for maintaining a healthy skin barrier and protecting against aging, as it delivers antioxidants such as vitamin E and coenzyme Q10 to the skin surface (Sakuma and Maibach, 2012; Smith and Thiboutot, 2008). However, excessive sebum secretion can negatively impact skin appearance by contributing to shine, clogged pores, and acne development. Z0010M probe measurements confirmed a significant reduction in sebum levels on participants’ cheeks. These results indicate that the cream containing VHProbi^®^ MixP is associated with reduced sebum excretion.

Porphyrins are metabolic byproducts generated during the bacterial catabolism of sebum (Barnard et al., 2020; Shu et al., 2013). Both qualitative and quantitative analyses confirmed a decline in porphyrin levels, consistent with reduced sebum content. Enlarged pore size is typically associated with excessive sebum production, as enlarged sebaceous glands develop due to impaired desquamation and reduced epidermal cell turnover (Roh et al., 2006). Zheng et al. investigated the relationship between facial skin types and bacterial composition in young Chinese females and found that pore size and density were significantly greater in oily skin compared to normal or dry skin (Zheng et al., 2021). Our findings regarding the pore index are consistent with these observations.

Imbalances and elevated lipid levels may lead to increased TEWL and reduced hydration. Zhang et al. reported significantly higher TEWL and significantly lower hydration levels in individuals with mild to severe oily sensitive skin compared to healthy controls (Zhang et al., 2024). Jiang similarly demonstrated that TEWL was significantly elevated and stratum corneum hydration was significantly reduced in individuals with oily sensitive skin relative to healthy individuals (Jiang et al., 2024). Marcella Gabarra et al. conducted a clinical trial and identified a correlation between high sebum levels and decreased stratum corneum water content (Gabarra Almeida Leite and Maia Campos, 2020). These findings collectively describe a well-documented phenomenon in skin physiology. In the present study, reduced sebum levels on participants’ cheeks were associated with improved skin barrier integrity, leading to enhanced water retention and restoration of the skin’s oil-to-water balance. Overproduction of sebum and enlarged pores can promote comedone formation, thereby increasing the risk of acne, blackheads, and seborrheic dermatitis. De Melo evaluated mature oily skin using microrelief analysis and observed increased skin texture values, indicative of surface roughness (de Melo and Maia Campos, 2018). We found that *L. paracasei* E12, one of the three strains used to produce VHProbi^®^ MixP, exhibited antioxidant activity by scavenging reactive oxygen species (ROS) in our previous study. Oxidative stress is known to enhance sebaceous gland activity and promote lipid peroxidation, which contributes to skin inflammation and the characteristic greasy appearance of oily skin. The antioxidant components in the ferment lysate may therefore reduce sebum secretion by mitigating oxidative stress in sebaceous glands. It is might reasonable to conclude that the cream containing VHProbi^®^ MixP is associated with ameliorated excessive sebum excretion, thereby providing multiple benefits, including improved hydration, reduced pore visibility, and smoother skin texture.

Sebum is a major nutrient source for lipophilic bacteria, particularly Cutibacterium. The observed decrease in sebum levels likely created a less favorable environment for the over proliferation of these taxa, thereby relieving the competitive pressure on other commensal microbes. Consequently, this ecological transition resulted in a notable increase in microbial diversity and a more balanced community structure.

This study suggests that regular use of the investigational skincare product may modulate the microbial community on cheek skin. The Shannon and Simpson indices showed changes following product application, with increased evenness and richness observed at Day 60 compared to Day 0, which aligns with previous findings by Zheng et al., who reported that oily skin in young Chinese females is associated with lower bacterial diversity and richness compared to dry or normal skin. Unlike the nutrient-rich environment of the human gut, the skin surface is limited in essential nutrients, consisting primarily of proteins and lipids, thereby constituting an oligotrophic habitat. On oily skin, lipophilic bacteria tend to proliferate excessively, leading to reduced microbial α-diversity (Li et al., 2017). Supporting evidence from *β*-diversity analysis indicated that a subtle increase in microbial community variability after treatment. Increased microbial diversity is widely regarded as a hallmark of a healthier skin microbiota (Prescott et al., 2017). Zhang et al. reported significant negative correlations between sebum secretion and α-diversity indices, contrasting with our findings (Zheng et al., 2021). This discrepancy may be attributed to differences in study population characteristics, including average age, sex, ethnicity, and intervention duration. Therefore, we hypothesize that the cream containing VHProbi^®^ MixP may be associated with modulated cutaneous microbial homeostasis in individuals with oily skin.

Species difference analyses revealed that the genera *Staphylococcus*, *Cutibacterium*, and *Streptococcus* might be the drivers of bacterial community changes in oily skin following 60 days of investigational cream application. Combined with Spearman correlation results, we observed that the decreased abundance of *Cutibacterium* was associated with an increase in *Lactobacillus*, consistent with findings from microbial composition analysis. *Cutibacterium* possess the ability to hydrolyze triglycerides, leading to the production of free fatty acids that exhibit broad-spectrum antimicrobial activity (Akaza et al., 2014). It is plausible that the negative correlation between *Cutibacterium* and *Lactobacillus* arises from antagonistic interactions mediated by these metabolites. Besides, our earlier *in vitro* studies confirmed that *L. plantarum* E15 exhibited specific inhibitory effects against *C. acnes* ATCC 11827 and ATCC 6919, a major lipophilic bacterium associated with excessive sebum production and acne development. The observed reduction in *Staphylococcus* spp. and *Cutibacterium* spp. and enrichment of *Streptococcus* spp. might be attributed to the selective antimicrobial activity of VHProbi^®^ MixP.

In this study, no significant correlations were observed between skin parameters and α- diversity, which contrasts with findings reported by Zheng et al (Zhai et al., 2018). Skin microbiota diversity and skin parameters change dynamically over time; therefore, single-time-point assessments may fail to capture transient or evolving relationships, potentially explaining the absence of observed correlations. Within the skin microbial ecosystem, certain keystone species may exert disproportionate influence on skin condition. The Spearman correlation heatmap indicated that the reduction in *Staphylococcus* abundance may be attributable to decreased sebum secretion, subsequently creating favorable conditions for *Streptococcus* enrichment. Previous studies have shown that *Cutibacterium* is the predominant bacterium in sebaceous glands due to its lipophilic nature, followed by *Staphylococcus*. Conversely, the abundance of *Streptococcus* increases with higher hydration levels and lower sebum levels (Debadatta, 2023; “Structure, Function and Diversity of the Healthy Human Microbiome,” 2012). Mukherjee et al. demonstrated that facial skin microbiota composition and diversity are jointly influenced by site-specific sebum levels and hydration status (Mukherjee et al., 2016). We may conclude that VHProbi^®^ MixP cream is associated with shifts in *Staphylococcus* abundance, resulting in a microbial profile consistent with reduced sebum level.

The ability of fermented lysate to enhance the skin’s natural repair processes and strengthen its resilience to environmental stressors renders it highly valuable in skincare formulations. With increasing consumer focus on achieving healthy, radiant skin, the demand for anti-oil skincare products containing fermented lysate has experienced substantial growth (Debadatta, 2023). However, this study has several limitations. First, the absence of a control group limits the strength of causal inference. Second, the potential placebo effect cannot be ruled out due to the unblinded design. Additionally, the specific bioactive compounds responsible for reducing excessive sebum production have not been identified. Furthermore, the sequencing depth of the skin microbiota samples was relatively shallow, which may restrict the detection of low-abundance taxa and affect the resolution of microbial community dynamics.

## 5 Conclusion

The findings from this exploratory single-arm, unblinded pilot study indicate that topical application of the cream containing fermented lysate VHProbi^®^ MixP is associated with ameliorated skin oiliness in the study cohort, alongside observed reductions in sebum secretion and modulated cutaneous microbial community structure over a 60-day application period. However, this study lacks a vehicle or placebo comparator group, which precludes the establishment of definitive causal relationships between VHProbi^®^ MixP and the observed improvements in oily skin parameters; additionally, the unblinded design cannot rule out potential placebo effects or participant bias. Therefore, well-powered, double-blind, placebo-controlled randomized trials are essential to validate the potential efficacy of VHProbi^®^ MixP, rule out confounding factors, and confirm the observed associative effects. Furthermore, detailed strain-level analysis of the skin microbiota is required to determine whether specific shifts in cheek skin bacterial populations are linked to VHProbi^®^ MixP application, and identification of the bioactive components within VHProbi^®^ MixP is needed to elucidate the potential mechanisms underlying the observed associations with improved oily skin status. Such follow-up studies must validate the preliminary findings of this pilot investigation and provide a more robust evidence base for the potential use of VHProbi^®^ MixP as a cosmetic ingredient for the management of facial seborrhea.

## Data Availability

The datasets presented in this study can be found in online repositories. The names of the repository/repositories and accession number(s) can be found in the article/**Supplementary Material**.
