# Supplementary material for: Evaluation of lactic acid ferment lysate for the management of oily skin: impact on sebum, hydration, and microbiota
Source: Front Physiol. 2026 Jun 5;17:1791680. doi: 10.3389/fphys.2026.1791680 (PMC13278995; doi:10.3389/fphys.2026.1791680)
Supplement: Supplementary file 1 [file DataSheet1.docx]

Table S1. Characteristics of the study subjects

| **Characteristics** | **Subjects (n=42)** |
| --- | --- |
| Age, years | 29.40±7.11 |
| Gender |  |
| Men, n (%) | 21 (50.0%) |
| Women, n (%) | 21 (50.0%) |

Note: Values are mean ± SD, number (percentage).

Table S2. Detailed scores of skin parameters for individuals from day 0 to day 60

|  | Sebum | | | Porphyrin | | | Pore | | | TWEL | | | Hydration | | | Texture | | |
| --- | --- | --- | --- | --- | --- | --- | --- | --- | --- | --- | --- | --- | --- | --- | --- | --- | --- | --- |
| No. | D0 | D30 | D60 | D0 | D30 | D60 | D0 | D30 | D60 | D0 | D30 | D60 | D0 | D30 | D60 | D0 | D30 | D60 |
| **S01** | 65 | 50 | 36 | 19.52 | 6.16 | 5.69 | 19.29 | 18.22 | 13.63 | 7 | 5 | 4 | 39.70 | 48.00 | 49.30 | 14.25 | 11.22 | 8.62 |
| **S02** | 66 | 60 | 43 | 12.79 | 3.09 | 1.04 | 10.00 | 10.65 | 9.12 | 5 | 2 | 1 | 43.70 | 38.00 | 41.30 | 4.91 | 3.57 | 2.36 |
| **S03** | 78 | 64 | 56 | 21.52 | 17.06 | 12.19 | 34.09 | 27.39 | 24.04 | 8 | 6 | 5 | 28.30 | 37.30 | 40.30 | 17.77 | 13.10 | 11.47 |
| **S04** | 68 | 63 | 54 | 25.28 | 9.18 | 7.94 | 32.68 | 29.41 | 22.12 | 5 | 4 | 4 | 22.70 | 33.70 | 36.70 | 22.01 | 17.19 | 16.01 |
| **S05** | 56 | 49 | 46 | 21.59 | 10.49 | 10.27 | 18.51 | 15.88 | 12.94 | 5 | 5 | 3 | 31.00 | 31.00 | 40.00 | 23.92 | 15.16 | 15.81 |
| **S06** | 60 | 41 | 31 | 32.70 | 22.86 | 18.46 | 32.00 | 31.12 | 25.90 | 4 | 3 | 1 | 13.70 | 19.70 | 27.00 | 9.19 | 8.66 | 7.90 |
| **S07** | 77 | 69 | 48 | 26.27 | 25.61 | 20.39 | 24.82 | 19.48 | 13.81 | 5 | 5 | 5 | 15.30 | 24.70 | 30.00 | 15.19 | 14.29 | 11.22 |
| **S08** | 60 | 55 | 41 | 18.01 | 10.71 | 6.77 | 13.70 | 9.61 | 8.80 | 7 | 3 | 2 | 25.70 | 25.00 | 33.00 | 2.56 | 1.19 | 0.98 |
| **S09** | 50 | 45 | 33 | 24.83 | 18.46 | 15.73 | 14.53 | 11.99 | 10.49 | 5 | 4 | 2 | 21.00 | 29.00 | 56.30 | 2.85 | 1.79 | 1.09 |
| **S10** | 73 | 51 | 49 | 20.15 | 19.90 | 19.19 | 11.78 | 9.86 | 7.20 | 6 | 6 | 4 | 35.00 | 51.00 | 52.00 | 3.58 | 2.50 | 1.98 |
| **S11** | 62 | 58 | 42 | 19.23 | 13.91 | 7.81 | 20.08 | 16.44 | 13.72 | 11 | 12 | 7 | 27.00 | 38.00 | 52.00 | 18.41 | 14.90 | 9.86 |
| **S12** | 70 | 62 | 43 | 10.45 | 8.82 | 7.93 | 17.47 | 16.49 | 15.59 | 9 | 7 | 6 | 35.30 | 45.00 | 64.30 | 5.37 | 2.23 | 1.61 |
| **S13** | 54 | 48 | 38 | 35.77 | 21.68 | 18.85 | 10.07 | 13.32 | 9.59 | 8 | 8 | 6 | 11.00 | 34.70 | 36.00 | 6.59 | 3.08 | 3.88 |
| **S14** | 63 | 50 | 45 | 21.77 | 15.32 | 13.43 | 22.47 | 19.63 | 16.37 | 6 | 5 | 4 | 54.70 | 57.00 | 71.30 | 13.84 | 12.52 | 7.55 |
| **S15** | 70 | 58 | 40 | 8.48 | 6.05 | 2.24 | 7.86 | 7.55 | 5.63 | 5 | 4 | 1 | 23.30 | 50.70 | 63.30 | 7.34 | 4.96 | 3.64 |
| **S16** | 71 | 58 | 43 | 19.22 | 13.15 | 7.76 | 26.80 | 21.70 | 19.97 | 5 | 4 | 4 | 36.70 | 57.70 | 64.30 | 9.00 | 5.62 | 5.61 |
| **S17** | 73 | 64 | 49 | 22.29 | 14.52 | 12.99 | 14.95 | 11.59 | 9.49 | 8 | 7 | 6 | 12.70 | 17.70 | 40.00 | 6.03 | 4.21 | 2.84 |
| **S18** | 76 | 61 | 58 | 26.85 | 20.87 | 12.18 | 22.14 | 26.16 | 18.97 | 5 | 5 | 3 | 38.00 | 40.30 | 20.30 | 21.26 | 18.03 | 15.36 |
| **S19** | 74 | 52 | 44 | 29.73 | 23.78 | 21.48 | 20.82 | 20.33 | 18.50 | 8 | 6 | 5 | 38.70 | 41.30 | 44.00 | 9.64 | 8.44 | 7.88 |
| **S20** | 62 | 48 | 38 | 17.68 | 10.36 | 8.34 | 15.62 | 13.33 | 12.12 | 5 | 4 | 2 | 33.00 | 35.00 | 60.00 | 3.11 | 2.38 | 2.12 |
| **S21** | 58 | 45 | 42 | 20.09 | 15.16 | 10.46 | 17.62 | 16.77 | 14.62 | 7 | 6 | 4 | 31.70 | 42.00 | 44.30 | 9.17 | 5.15 | 5.07 |
| **S22** | 50 | 41 | 37 | 16.25 | 10.48 | 7.18 | 15.18 | 13.24 | 11.05 | 5 | 3 | 2 | 29.70 | 38.70 | 47.00 | 14.36 | 15.50 | 10.53 |
| **S23** | 54 | 43 | 38 | 15.11 | 11.02 | 10.82 | 10.66 | 8.99 | 8.34 | 5 | 3 | 2 | 21.70 | 56.30 | 59.70 | 5.10 | 2.16 | 1.76 |
| **S24** | 63 | 49 | 41 | 19.09 | 14.88 | 12.35 | 24.73 | 18.31 | 17.08 | 9 | 8 | 7 | 23.00 | 36.00 | 41.30 | 19.33 | 16.63 | 11.70 |
| **S25** | 55 | 49 | 46 | 16.12 | 10.75 | 6.49 | 16.77 | 15.69 | 14.33 | 7 | 10 | 5 | 20.30 | 28.70 | 33.30 | 21.74 | 18.38 | 12.56 |
| **S26** | 64 | 54 | 45 | 11.16 | 7.03 | 4.05 | 13.98 | 12.36 | 10.02 | 8 | 5 | 3 | 35.00 | 44.30 | 56.00 | 12.90 | 11.10 | 10.51 |
| **S27** | 58 | 50 | 40 | 19.38 | 16.91 | 12.33 | 15.35 | 12.00 | 12.51 | 5 | 3 | 2 | 12.30 | 34.70 | 46.30 | 8.67 | 7.17 | 4.01 |
| **S28** | 73 | 46 | 44 | 15.66 | 6.49 | 5.89 | 31.61 | 27.41 | 26.79 | 4 | 4 | 2 | 38.00 | 43.30 | 61.70 | 6.58 | 3.66 | 4.55 |
| **S29** | 66 | 46 | 41 | 12.87 | 7.92 | 7.08 | 11.12 | 10.37 | 9.79 | 6 | 5 | 3 | 30.00 | 41.70 | 48.70 | 2.17 | 2.05 | 1.78 |
| **S30** | 58 | 46 | 48 | 20.46 | 16.10 | 12.77 | 11.77 | 7.47 | 7.84 | 8 | 7 | 6 | 24.00 | 37.00 | 46.30 | 18.14 | 7.02 | 8.94 |
| **S31** | 58 | 38 | 35 | 9.90 | 7.74 | 7.80 | 20.47 | 21.47 | 17.28 | 7 | 4 | 3 | 45.30 | 48.00 | 52.00 | 8.20 | 6.76 | 5.34 |
| **S32** | 60 | 45 | 37 | 11.97 | 8.24 | 8.00 | 14.78 | 14.78 | 10.24 | 5 | 5 | 2 | 41.30 | 41.00 | 65.00 | 7.02 | 6.41 | 4.24 |
| **S33** | 66 | 57 | 48 | 24.50 | 19.66 | 16.82 | 16.43 | 13.95 | 13.84 | 6 | 6 | 4 | 32.30 | 41.00 | 51.00 | 13.57 | 12.82 | 5.73 |
| **S34** | 62 | 49 | 45 | 10.46 | 7.60 | 7.64 | 22.26 | 14.26 | 12.66 | 5 | 3 | 1 | 28.70 | 37.30 | 64.70 | 7.35 | 5.23 | 4.29 |
| **S35** | 75 | 64 | 57 | 19.16 | 14.73 | 11.94 | 29.21 | 26.83 | 21.37 | 7 | 4 | 3 | 14.00 | 25.30 | 30.00 | 18.04 | 14.72 | 13.85 |
| **S36** | 51 | 40 | 34 | 11.67 | 6.51 | 5.51 | 24.95 | 25.19 | 21.41 | 7 | 4 | 4 | 54.30 | 59.00 | 61.30 | 8.46 | 5.22 | 4.51 |
| **S37** | 66 | 58 | 52 | 15.46 | 10.93 | 9.61 | 51.72 | 45.44 | 46.03 | 8 | 5 | 4 | 28.30 | 41.70 | 49.70 | 20.56 | 16.99 | 12.99 |
| **S38** | 74 | 56 | 51 | 17.14 | 11.38 | 9.03 | 8.70 | 6.32 | 4.36 | 8 | 7 | 7 | 46.70 | 47.30 | 53.00 | 4.20 | 2.31 | 1.55 |
| **S39** | 67 | 49 | 42 | 9.17 | 3.64 | 2.64 | 8.42 | 6.00 | 5.48 | 10 | 6 | 6 | 15.70 | 32.70 | 35.00 | 2.63 | 1.32 | 0.75 |
| **S40** | 65 | 62 | 58 | 15.97 | 10.87 | 10.59 | 22.49 | 17.40 | 15.47 | 8 | 6 | 5 | 30.30 | 46.00 | 63.30 | 4.68 | 4.00 | 5.04 |
| **S41** | 60 | 57 | 46 | 53.57 | 48.38 | 29.61 | 14.10 | 11.23 | 11.55 | 4 | 4 | 3 | 14.00 | 33.70 | 44.00 | 13.05 | 5.43 | 4.04 |
| **S42** | 57 | 40 | 39 | 13.46 | 9.81 | 10.79 | 17.08 | 16.44 | 6.40 | 10 | 8 | 8 | 37.70 | 50.30 | 55.70 | 5.51 | 5.15 | 4.42 |

D0: day 0; D30: day 30; D60: day 60;

Table S3. **The results of efficacy** **Self-assessment Questionnaire for oily skin**

| Please answer efficacy-related questions below | Not at all | Slightly | Some | Good | Excellent |
| --- | --- | --- | --- | --- | --- |
| Do you feel your skin less greasy? | 0(0.0%) | 0(0.0%) | 0(0.0%) | 6(14.3%) | 36(85.7%) |
| Do you feel your skin smoother? | 0(0.0%) | 0(0.0%) | 0(0.0%) | 3(7.1%) | 39(92.9%) |
| Do you feel your skin finer? | 0(0.0%) | 0(0.0%) | 0(0.0%) | 4(9.5%) | 38(90.5%) |
| Do you feel your skin less itchy? | 0(0.0%) | 0(0.0%) | 0(0.0%) | 8(19.0%) | 34(81.0%) |
| Do you find your facial pores less and(or) smaller? | 0(0.0%) | 0(0.0%) | 0(0.0%) | 10(23.8%) | 32(76.2%) |
| Does your skin become more stable with less chance showing breakouts, closed pores, keratic plugs or inflammation, etc.? | 0(0.0%) | 0(0.0%) | 0(0.0%) | 6(14.3%) | 36(85.7%) |
| Do you feel your skin with better tone? | 0(0.0%) | 0(0.0%) | 0(0.0%) | 8(19.0%) | 34(81.0%) |
| Do you find improvement of your skin condition? | 0(0.0%) | 0(0.0%) | 0(0.0%) | 1(2.4%) | 41(97.6%) |

Note: Values are number (percentage).


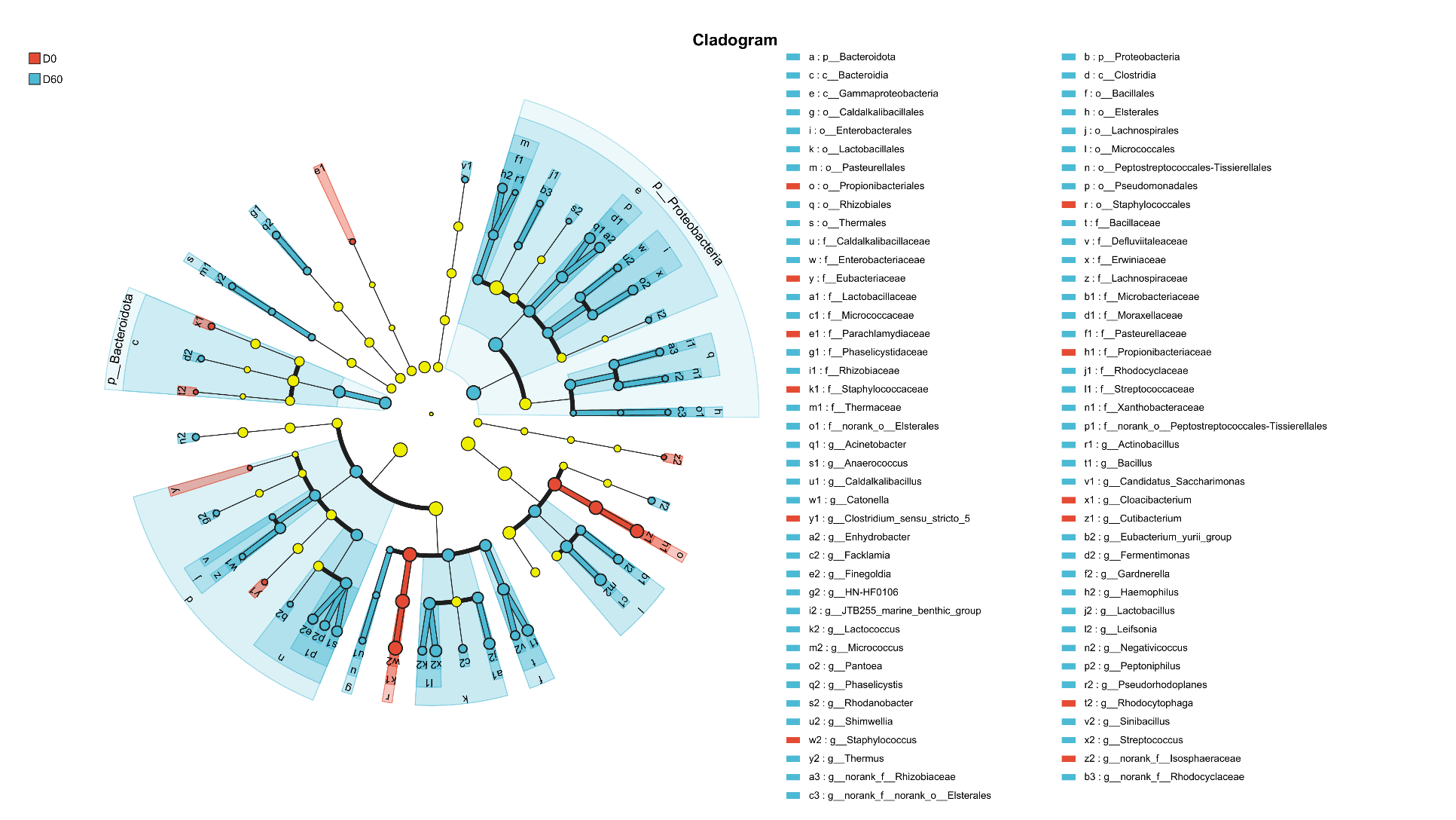


**Figure S1. Cladogram of LEfSe (LDA Effect Size) analysis.** Different color nodes represented microbial groups that were significantly enriched in the corresponding groups and had a significant effect on the differences between groups (LDA >2, p < 0.05), while yellow nodes represented microbial groups that have no significant difference in different groups or have no significant effect on the differences between groups (LDA <2). Red: D0 group; Cyan; D60 group; Only identifiable species with LDA >2 was shown. D0: day 0; D60: day 60;
